# Publisher’s Note to Volume 76, Supplement 2

**DOI:** 10.1093/jac/dkab502

**Published:** 2022-01-25

In the originally published versions of articles in Volume 76, Supplement 2 of *Journal of Antimicrobial Chemotherapy* (https://academic.oup.com/jac/issue/76/Supplement_2), roman numeral page number prefixes (ii) were omitted in error. This error has been corrected. The publisher apologizes for the error.

